# Current Hospital Topics

**Published:** 1906-05-12

**Authors:** 


### CURRENT HOSPITAL TOPICS.

### Value of Government Inspection of Hospitals.

One advantage of the system pursued in regard to the inspection of hospitals in Australasia was shown at a recent meeting of the managers of the Alfred Hospital, Melbourne. It was reported that the Government grant had been reduced by £200, because Mr. Short, the inspector of Charities, stated that the stay of each patient had of late exceeded twenty-five days, which the Government considered should be the maximum at a general hospital. Having regard to the marked tendency exhibited by hospitals generally to increase their expenditure per bed year by year, it is satisfactory to note that Government inspection is able in this way to enforce some economy, and to exercise control over the class of cases admitted to treatment and their retention in the hospital. It was decided to refer the matter to the medical staff for report, but in the meantime it was stated, in consequence of the Inspector's action, that the average stay of every in-patient during the current financial year had been reduced to less than twenty-five days, showing the effective influence exercised over the administration of hospitals by the Government.

### A Blow to Provident Dispensaries.

According to the *Birmingham Daily Post* something like consternation has been caused amongst the managing committees and members of provident dispensaries up and down the country, owing to the active steps being taken to put into force, with local modifications generally of an unimportant character, the recommendations of the medico-political committee of the British Medical Association. These recommendations were briefly: (1) a wage limit for benefiting members of such organisations; (2) higher rates of payment (in some cases) to the professional men holding appointments as medical officers; (3) the committees of management of provident dispensaries and other public medical services to have on them a clear majority (or at least half) of medical men; (4) all registered medical practitioners resident within the area of operation of any service to be eligible for appointment to the staff; (5) canvassing, advertising, and collection of payments forbidden. The three Midland towns in which provident dispensaries flourish most are Coventry, Leicester, and

Northampton, where the medical staffs of this class of institution have received considerable sums each year for medical attendance. Thus, in the year ending March 1906, the medical staff of the Coventry Provident Dispensary were paid £2,404 in fees out of a total income of £3,875. It is held locally that the interference of the British Medical Association will have the tendency to do more harm than good by disturbing a system which on the whole has worked satisfactorily to the public and the profession.

### Isolation Hospital Extravagances.

We have had occasion to call attention to the defective arrangements for the comfort and treatment of patients in isolation and infectious hospitals, especially in rural districts. An inquiry on behalf of the Local Government Board inspector, Dr. R. W. Johnstone, held at Harrogate, relative to the application to borrow £3,000 for the purpose of building and furnishing the Joint Isolation Hospital at Thistle Hill, near Knaresborough, revealed how little knowledge may be exhibited and economy practised by local authorities in regard to these hospitals The hospital contains forty-eight beds, and the total cost when completed is estimated at £25,000. This will represent an expenditure exceeding £520 per bed—an outrageous figure in the circumstances and one which may lead the Local Government Board to refuse its sanction to the further loan asked for by the County Council, as the increased expenditure seems due to the tenders having been exceeded, according to the report in the local papers. We will take three items as specimens of the want of intelligent method pursued by those responsible in the present case. One item is one of £40 for the erection of a commemorative tablet bearing the names of the Committee, etc. Is it surprising that the inspector expressed a doubt whether the Board would sanction this item? Thirty pounds, reduced to twenty-five in consequence of a criticism, was expended upon a carpet to be placed over the linoleum laid down in the corridor. We are of opinion that this expenditure should not be sanctioned, and that the carpet should be removed as unnecessary, linoleum being admittedly the best covering for a hospital corridor. Again, six lounge chairs in crimson leather were purchased for the wards at a cost of twelve guineas. It further appeared that stone mantelpieces were fixed in the kitchens, one of which had been painted to imitate marble, a proceeding which must make it unsuitable for hospital purposes in a hygienic sense, apart from æsthetic considerations. We should be glad to see a copy of the report of the Local Government Board inspector as the result of his inquiries at Knaresborough, and to have his comment upon a system which advertises for tenders and accepts only the lowest in the majority of instances. This policy seems based upon the consideration mentioned by Mr. Wilson, the chairman, that in some cases orders were split up amongst the different tradespeople in the district The representatives of Knaresborough justly expressed indignation at the expenditure which had been sanctioned, for, as Mr. H. Eddy put it, it was not necessary to furnish a hospital in the lavish way a gentleman might furnish his house. Mr. H. Horspool, another Knaresborough representative, declared that much of the expenditure was quite unnecessary, and, had economy been observed, several hundreds of pounds could have been saved. There can be no doubt that the ratepayers' representatives should be stirred up to exercise more control in regard to the expenditure upon isolation hospitals all over the country

### A Hospital to be Supported.

One of the most interesting, and what we hope may prove one of the most successful of the season's charity dinners, will be presided over by H.R.H. the Duke of Connaught at the Grocers' Hall on May 15, in support of this hospital. Situated as it is in one of the poorest districts of London, out of sight of the more wealthy residents of the metropolis, it has not yet received the amount of support from those who are specially interested in children's hospitals which the character of the work and the quality of the management should undoubtedly secure for it. The hospital contains 125 beds in constant use, and the recent united effort on the part of the representatives of the various religious communities in the district where the hospital is situated, bears unique testimony to the urgency of its needs, and the value of the work, which it has so successfully carried on for many years, on behalf of the impoverished children who abound in its neighbourhood. The Bishop of Stepney, the Chief Rabbi, the Vicar of St. Michael's, London Fields, and the Chairman of the Clergy Emergency Committee have issued an earnest appeal on behalf of the Dinner Fund, and we hope that all who are interested in sick children will make a point of sending some contribution for the Duke of Connaught's list. Letters should be addressed to Mr. T. Glenton Kerr, Secretary, North-Eastern Hospital for Children, Hackney Road, Bethnal Green, E.

